# GEMA—An Automatic Segmentation Method for Real-Time Analysis of Mammalian Cell Growth in Microfluidic Devices

**DOI:** 10.3390/jimaging8100281

**Published:** 2022-10-14

**Authors:** Ramiro Isa-Jara, Camilo Pérez-Sosa, Erick Macote-Yparraguirre, Natalia Revollo, Betiana Lerner, Santiago Miriuka, Claudio Delrieux, Maximiliano Pérez, Roland Mertelsmann

**Affiliations:** 1 CONICET—National Scientific and Technical Research Council, Buenos Aires C1004, Argentina; 2 Faculty of Informatics and Electronic, ESPOCH—Polytechnic School of Chimborazo, Riobamba 060155, Ecuador; 3 IREN Center, UTN—National Technological University, Pacheco, Buenos Aires B1618, Argentina; 4 Department of Electrical and Computer Engineering, USN (National University of the South), Bahía Blanca B8000, Argentina; 5 Department of Electrical and Computer Engineering, FIU (Florida International University), Miami, FL 33174, USA; 6 Faculty of Engineering, Institute of Engineering Biomedical, UBA (University of Buenos Aires), Buenos Aires C1053, Argentina; 7 Collaborative Research Institute Intelligent Oncology, Hermann-Herder-Straße 4, 79104 Freiburg im Breisgau, Germany; 8 LIAN-CONICET-FLENI—Foundation for the Fight against Childhood Neurological Diseases, Belén de Escobar B1625, Argentina; 9 Department of Medicine I, Medical Center, Faculty of Medicine, University of Freiburg, 79085 Freiburg im Breisgau, Germany

**Keywords:** biological image segmentation, apoptosis process, linear regression, real-time analysis

## Abstract

Nowadays, image analysis has a relevant role in most scientific and research areas. This process is used to extract and understand information from images to obtain a model, knowledge, and rules in the decision process. In the case of biological areas, images are acquired to describe the behavior of a biological agent in time such as cells using a mathematical and computational approach to generate a system with automatic control. In this paper, MCF7 cells are used to model their growth and death when they have been injected with a drug. These mammalian cells allow understanding of behavior, gene expression, and drug resistance to breast cancer. For this, an automatic segmentation method called GEMA is presented to analyze the apoptosis and confluence stages of culture by measuring the increase or decrease of the image area occupied by cells in microfluidic devices. In vitro, the biological experiments can be analyzed through a sequence of images taken at specific intervals of time. To automate the image segmentation, the proposed algorithm is based on a Gabor filter, a coefficient of variation (CV), and linear regression. This allows the processing of images in real time during the evolution of biological experiments. Moreover, GEMA has been compared with another three representative methods such as gold standard (manual segmentation), morphological gradient, and a semi-automatic algorithm using FIJI. The experiments show promising results, due to the proposed algorithm achieving an accuracy above 90% and a lower computation time because it requires on average 1 s to process each image. This makes it suitable for image-based real-time automatization of biological lab-on-a-chip experiments.

## 1. Introduction

Over the past few years, microscopy has been an important technique to study biological environments. Recent advancements in high-resolution fluorescent and brightfield microscopy have been of great importance for detailed visualization of the cells and their subcellular structures. Moreover, improvements in automated microscopy such as high-content screening and high-performance time-lapse, enable automatically computing of phenotypic changes of cells under a wide array of dissimilar environmental conditions and obtaining a better understanding of biological function through the study of cellular dynamics, respectively [1].

Microfluid devices have automated microscope hardware to parallelize experiments to both increase data resolution and test countless biological conditions [2]. Lab-on-a-chip technology has been broadly accepted by biological and medical scientific communities as a promising tool for the control of the microenvironment at the molecular, cellular, and tissue levels [3]. Due to the amount of data from microfluidic chips, it is necessary to develop new algorithms to allow the analysis of data and images with a high performance and efficient use of computational resources.

In [4], an image processing software, Python based image analysis for cell growth (PIACG) has been developed, that is able to calculate the total area of the well occupied by cells with fusiform and rounded morphology in response to different concentrations of fetal bovine serum in microfluidic chips, from microscopy images in transmission light, in a highly efficient way. The software is able to segment and quantify images with diverse cellular densities and different cellular morphology automatically, without parameter adjustment and considerable time-saving in data analysis, through a simple and sensible user interface, providing in a quick and simple way a multitude of statistical data.

Analyzing and developing different methods could be relevant in the research of future treatments against diseases such as breast cancer. Breast cancer is the most common malignancy in women around the world [5]. In quantitative image-based cell biology, cellular assessments are mainly derived from detected objects applying image segmentation methods. This requires automated segmentation which includes algorithm design, evaluation, and computational scalability in high-throughput applications [6].

With this aim, several automatic segmentation algorithms have been developed to work with phase contrast and differential interference contrast data. The most common approaches involve thresholding, active contours, and Watershed techniques [7]. Software packages commercial have also been developed to compound many of these ordinary approaches, suitable across various cell lines and modalities.

In [8], CellProfiler works adequately only for reconstructed images. In [9], ImageJ is open source software for processing and analyzing scientific images. This tool is developed in Java. In [10], BioImageXD is a multi-purpose post-processing tool for bioimaging. BioImageXD (version 1.0) was published in the July 2012 issue of Nature Methods. In [11], Icy is an open community platform for bioimage informatics to visualize, annotate and quantify bioimaging data. In [12], FIJI is a distribution of ImageJ which includes many useful plugins contributed by the community. This tool is used for biological image processing. In [13], FarSight was fruitless with the automatic threshold during foreground segmentation. In [14], Fogbank has been successfully applied to image segmentation using phase contrast, bright field, and fluorescence microscopy and binary images.

At the same time, due to the difficulty in accessing a large amount of labeled data, semi-supervised learning is becoming an attractive solution in medical image segmentation. Their success heavily depends on a large amount of annotated data which are hard to be available in the real world, especially in the field of medical image segmentation due to their expensive and time-consuming nature.

In [15], to diminish the limitation caused by label scarcity, a tripled-uncertainty guided semi-supervised model for medical image segmentation was proposed, which can effectively utilize the unlabeled data to improve the segmentation performance. Based on a mean teacher architecture, this model explores the relationship between the segmentation task, the foreground and background reconstruction task, and the signed distance field prediction task. The experimental results on two public medical datasets have demonstrated the feasibility and superiority of this method.

In [16], FastER was unsuccessful as a consequence of the nature of its maximally stable extremal region (MSER) detector. In [17,18,19], the segmentation algorithms—CellStar, SuperSegger, CellSerpent—were completely inappropriate for label-free non-round adherent cells with Dice coefficient < 0.1 [20]. Additional algorithms for segmentation are presented in [21]. However, it is necessary to improve the performance of segmentation algorithms when a specific application is aborded like biological experiments.

In this case, an advanced machine learning-based algorithms employ image gradients and pixel traces to enhance threshold values, but it still needs the criteria of the user as input to select a background elimination factor according to a dataset [22]. On the other hand, most deep learning-based methods output a probability map and use a hand-crafted threshold to generate the final segmentation result, while how confident the network is of the probability map remains unclear.

In [23], an uncertainty-guided network called UG-Net for automatic medical image segmentation was proposed. This network can learn from and contend with uncertainty by itself in an end-to-end manner. Experimental results show that the proposed network outperforms the state-of-the-art methods for nasopharyngeal carcinoma (NPC) segmentation, lung segmentation, optic disc segmentation, and retinal vessel detection. Although deep learning is currently dominant in medical image segmentation, there is no unified network due to the different target sizes of different tasks.

According to the state of art, most segmentation algorithms could fail under different circumstances which include the variance of contrast, luminescence, and cell densities when they approximate the confluency stage [24]. Unluckily, microscope image analysis remains a bottleneck for image segmentation. While plenty of software packages and algorithms have been developed focused on biological cells, the majority of them crave manual inputs from the experimenter and are designed for further processing which limits both accuracy and performance. Another issue in image analysis is related to real-time applications. It depends on the available resources, both experimentally and computationally. In this sense, it is necessary to develop algorithms capable of processing images optimally and adapted to the environment of biological experimentation.

**Figure 1:**
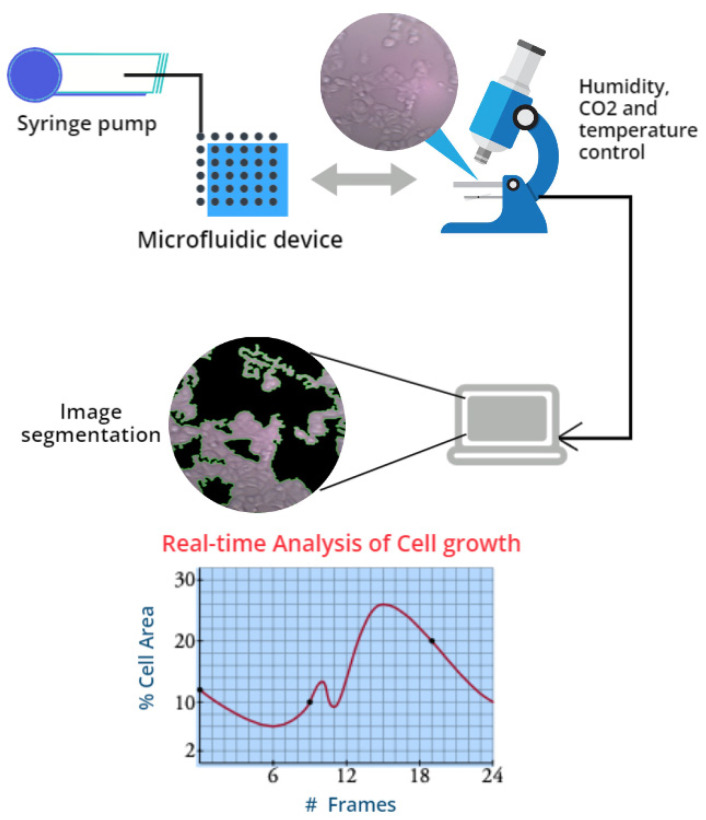
The computational system and hardware devices used in biological experiments.

In this paper, a real-time automatic cell segmentation method called GEMA (Gabor filter in biological experiments with morphology operations for segmentation applications) is proposed. In Figure 1, a general description of the computational system and hardware devices used to analyze the behavior of mammalian cells during experiments is presented.

To overcome the challenges mentioned previously, GEMA is a real-time automatic segmentation method for cell segmentation that uses linear regression to compute the optimal parameters to binarize images using an adaptive threshold method. In addition, linear regression allows to describe and predict the behavior of the experiment according to the image sequence through the computation of the percentage of area covered by cells in each image. This percentage of area represents the evolution of biological agents in time. Evolution is analyzed in two main stages: apoptosis and confluency [25,26].

This paper is organized as follows: Section 2 shows the biological framework. In this section, a description of the MCF7 cells with the different parameters used during experiments is presented. Section 3 shows the proposed method. In the first part, the mathematical description and pseudocode of GEMA are presented. In the second part, three relevant methods used to segment images such as manual segmentation, morphological gradient, and semi-automatic algorithms are presented. These methods are used to compare their performance in the segmentation process of biological images. Section 4 shows the experimental results obtained in the analysis and segmentation of two biological datasets. Moreover, the results are compared to evaluate the performance and accuracy of GEMA in front of the three algorithms. Section 5 shows the discussion about these experimental results, conclusions, and future work.

## 2. Biological Framework and Materials

The biological framework is focused on analyzing and developing different methods which could be relevant in the research of future treatments against diseases such as breast cancer. This kind of cancer is the most common malignancy in women around the world [5,27].

### 2.1. Cell Line—MCF7

Over the last few years, breast cancer has become a critical public health problem. In order to research this disease in depth, several cell lines have been designed for in vitro studies. This allows understanding behavior, gene expression, and drug resistance, among other variables [28].

One of these cell lines is MCF7 (Michigan Cancer Foundation-7), which has been extensively studied for 40 years. This type of cell has been studied in both in vitro and in vivo systems, to understand their cell-to-cell interaction, their metabolism, angiogenesis, etc. [29]. However, the maintenance of this cell line is inexpensive and does not usually require extra factors to maintain its characteristics. In addition, it has shown great stability along cell passages. This allows its growth to be homogeneous and the repeatability of the experiments to be plausible, while another point to be noted is its resistance to drugs that make the line candidate for tests with different drugs such as the apoptosis inducer used in this study, camptothecin (CPT) [30].

### 2.2. Apoptosis of the MCF7

The CPT drug was used to induce apoptosis of the MCF7 cell line. It was dissolved in the medium at concentrations 1 to 5X and with exposure times of 10, 18, and 24 h. The injection was carried out with flows of 250, 275, and 300 μL/min testing cell behavior in front of different drug concentrations.

Another important feature to note is that these cells are adaptable to various surfaces, both adhesive plastic and glass without the need for a pre-surface treatment. This makes the MCF7 cell line very versatile, especially for the present study where microdevices with a glass surface without adherent treatment were used.

### 2.3. Design and Fabrication of Microfluidic Devices

According to the Olmos et al., the microdevices were designed with Layout Editor software and transferred to TIL with a 2400 PPI infrared source; then TIL was laminated onto an unexposed photopolymer plate, photopolymer plate was exposed to UVA light at 0.45 J at the back for 10 s, a part of the photopolymer was covered with a mask plate at the back, and the photopolymer plate was exposed to 0.45 J UVA light at the back for 20 s. The above exposures were repeated one at a time.

In the next step, the front part was exposed to UVA light at 19 J for 360 s. After removing the TIL, the plate was washed with PROSOL N-1 solvent (supplied by Eastman Kodak) at 360 mm min^−1^ was dried in an oven for 30 min. at 50 °C, the plate had a last exposure to UVC light at 10 J for 17 min., and UVA light was applied at 4 J for 2 min. on the cold side with the mold manufactured briefly, a mixture of epoxy resin and curing agent (Crystal -T ack, Novarchem, Villa Martelli, Argentina) on the female mold to replicate the high relief design.

**Figure 2:**
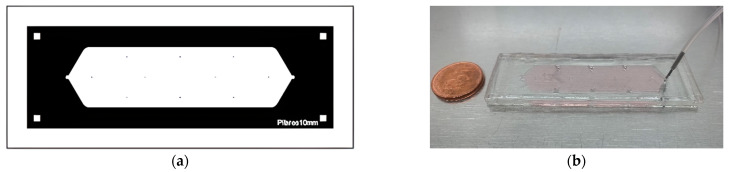
The PDMS device used during experiments. (**a**) Design view; (**b**) Physical view.

After curing, the epoxy resin mold (ERmold) was separated from the Fmold to form the male mold. Subsequently, a mixture of PDMS and curing agent in a 10:1 weight ratio (Sylgard 184 silicone elastomer kit, Dow Corning, Midland, MI, USA) was poured onto the ER mold and cured in an oven at 40 °C overnight [15]. In Figure 2, the final design and microfluid device PDMS used in the experiments are presented.

### 2.4. Cell Culture in Microdevices

The MCF7 cells were cultured in DMEN f12 Gibco basal medium, supplemented with 10% fetal bovine serum, 1% GlutaMAX and 1% Penicillin-Streptomycin Gibco as shown in Figure 2. The cell cultures were maintained at 37 °C in a saturated atmosphere with 95% of air and 5% of ${\mathrm{CO}}_{2}.$

A total of 100,000 cells were seeded per mL, with flows of 300 μL/min he total volume 140 injected in each device was 500 μL. Cells were cultured within the microdevices for up to 7 days. The change of medium was performed every 24 h, to stimulate growth exponentially, prior to the induction of apoptosis by CPT.

According to these characteristics, the designed system and biological framework allowed for maintaining the cells in the long term. Therefore, it is necessary to automate some of the processes that are involved in the experiment. In general, a microscope catches an image in an interval of time. This generates an image sequence that is analyzed by computational algorithms to evaluate the state of the cell culture at a specific time.

## 3. Algorithms for Image Segmentation

According to the introduction, several methods have been developed for automatic segmentation using different techniques. In this work, GEMA is proposed as a novel approach for using in segmentation of biological images. This algorithm is designed to work in real time which is useful both to analyze the increase or decrease in the number of the cells and to describe the behavior in time of a biological experiment during the administration of different drugs.

### 3.1. Datasets and Software Description

GEMA is programmed in Python using Google Colaboratory (Colab) [31]. It is publicly available to facilitate distribution and adaptation by other researchers. The source code and examples can be found in [32]. Three different methods are compared with GEMA. Firstly, the gold standards are obtained from a manual segmentation by experts. Secondly, a segmentation method based on a morphological gradient operator. And finally, a semi-automatic method using FIJI which is a library widely used in biology.

In order to test the performance of algorithms, 1775 images were used which come from two different experiments called Dataset_1 and Dataset_2. These experiments are available in Kaggle repository [33]. Each dataset contains two different sequences of images: the first one during the growth of cell culture to achieve the confluency stage and the second one during the cell death when the CPT drug was injected. Datasets were acquired from 24 to 72 h.

### 3.2. Manual Segmentation for Gold Standard Images

Experts have performed a manual segmentation to separate the total area occupied by cells from the image background for obtaining the gold standard. These images have been used to validate the results obtained by GEMA and to compare them with gradient and semi-automatic methods.

**Figure 3:**
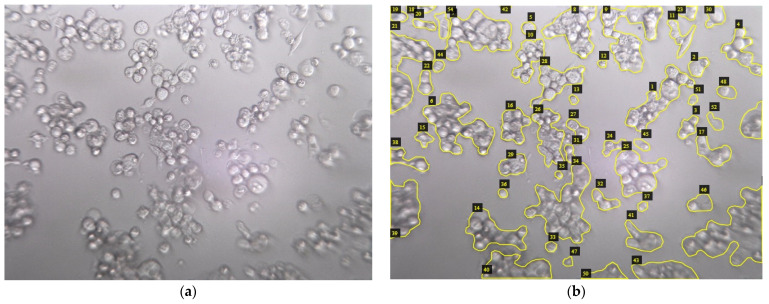
The MCF7 breast cancer cell line. (**a**) Image from Dataset 2—CPT_24H_72H; (**b**) manual segmentation of cell regions.

The VGG Image Annotator (VIA) program allowed the manual annotation to define the interest regions in images [34]. These regions can be described using geometrical shapes such as rectangles, circles, ellipses, polygons, etc. In this case, the polygon shape was used to select and enclose the cell regions as shown in Figure 3.

### 3.3. Algorithm Using FIJI for Semi-Automatic Segmentation

A semi-automatic method to quantitate cells by micropore was programmed using the macro developed by Rosero et al. [35] for the ImageJ platform version 1.49 (http://imagej.nih.gov/ij/ (accessed on 23 June 2021)) provided in the public domain by the National Institutes of Health, Bethesda, MD, USA, and coded the script using IJ1 programming language on Fiji image processing software (http://fiji.sc/ Fiji in the public domain (accessed on 23 June 2021)).

After file importation to the macro, the whole stack of the digital RGB images was converted to 8-bit. The convolve tool allows for increasing resolution of the image and brightness of each image to quantify cells. The matrix to increase the intensity used the center that corresponds to the source pixel and the other elements corresponding to neighboring pixels.

The destination pixel is calculated by multiplying each source pixel by its corresponding kernel coefficient and adding the results. Then, variance was used in the stack by replacing each pixel with the neighborhood. Finally, to quantify cells, the final stack was converted to binary in order to measure the percentage of cell area.

### 3.4. Gradient Operator for Automatic Segmentation

Morphological operators enhance the variation of pixel intensity in each neighborhood [36]. Each pixel of a gradient image measures the change in intensity of that same point in the original image. To segment the cancer cell areas, morphological gradient as gradient operators tend to enhance intensity variations on images is used.

The morphological gradient is defined as the *arithmetic* difference between the dilation and the erosion operation of an image with a structuring element B. The mathematical definition is presented in Equation (1).

(1) $$g\left(I\right)=\delta_{\left\{\rho B\right\}\left(I\right)}-\epsilon_{\left\{\rho B\right\}\left(I\right)}$$

where $\delta_{\left\{\rho B\right\}},\;\epsilon_{\left\{\rho B\right\}}$ are the dilation and the erosion operations, respectively.

The gradient image contains neighborhood-based information: the difference of a kernel of 5×5 pixel dilation and erosion. This difference is computed through the grayscale intensity I from RGB channels using $I=\frac{\left(R+G+B\right)}{3}$.

### 3.5. GEMA for Automatic Segmentation

Gradient method and GEMA have a preprocessing stage which is an important step in image analysis. Contrast limited adaptive histogram equalization (CLAHE) [37] and Gaussian filter for smoothing are used to improve the quality of the images.

**Figure 4:**
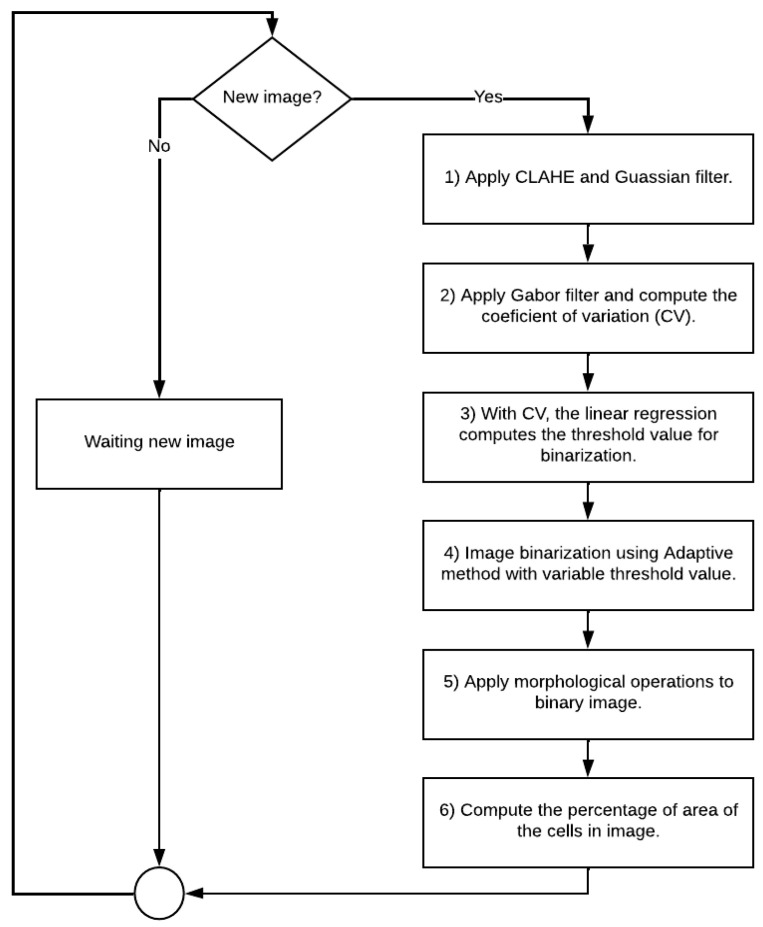
The GEMA flowchart used to segment MCF7 cells from image background.

The GEMA flowchart is presented in Figure 4 where the main processes are: (a) Gabor filtering to identify regions with the highest response. (b) Compute the coefficient of variation (CV) of pixels values in an image. This coefficient will be modeled through a linear regression. (c) Binary conversion using an Adaptive method with a variable parameter, and (d) Morphological operations are applied over the binary image to connect interest regions and/or delete non-interest regions.

#### 3.5.1. Gabor Filter Bank

The Gabor Filter has real and imaginary components representing orthogonal directions [38]. In two dimensions (2D), the Gabor filter is defined as shown in Equation (2):

(2) $$g\left(x,\;y\right)=\frac{f^{2}}{\pi \gamma \eta }\;exp-\left[\frac{x^{\prime }{}^{2}f^{2}}{\gamma^{2}}+\frac{y^{\prime }{}^{2}f^{2}}{\eta^{2}}\;\right]e^{-j2\pi f\;x^{\prime }}$$

where $x^{\prime }=x\;cos\theta +y\;sin\theta$, $y^{\prime }=-x\;sin\theta +ycos\theta \;.$ Furthermore x ,  y are the coordinates of frequencies in the reference system, $x^{\prime },\;y^{\prime }$ in θ orientation of Gabor function, f represents the distance from origin to center of Gaussian function, γ and η characterize the sharpness of Gaussian function along the major and minor axes.

**Figure 5:**
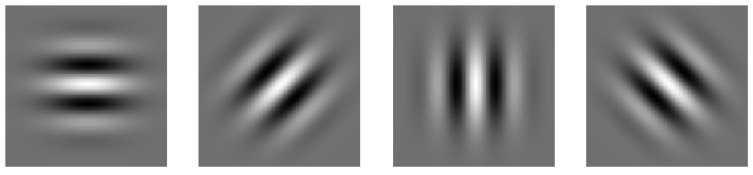
Parameters of the Gabor filter set: f=3.0, size=21, 4 rotations: $\theta_{0}=0^{{}^{\circ}}$, $\theta_{1}=45^{{}^{\circ}}$, $\theta_{2}=90^{{}^{\circ}},\;\theta_{3}=135^{{}^{\circ}}$ and γ=10, η=0.5.

A set of Gabor filters is used for detecting features in a given input image. This set can be computed in a space of M scales (frequencies) and N rotations to ensure invariance [39]. GEMA uses a set that is composed of 4 filters with 1 scale value and 4 different angles for rotation values. This set avoids the redundancy in features and optimizes the processing time. The real component of this set is presented in Figure 5.

#### 3.5.2. Coefficient of Variation and Linear Regression

The coefficient of variation is a standardized measure of the dispersion of a probability distribution or frequency distribution [40]. A mathematical definition for a population of data is presented in Equation (3):

(3) $$CV=\frac{\sigma }{\mu }$$

where *CV* is the coefficient of variation which describes the ratio of standard deviation σ to the mean μ.

**Figure 6:**
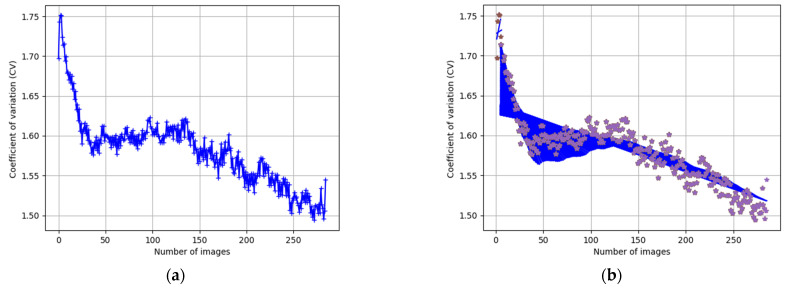
Dataset-1 with CPT for inducing apoptosis from confluency stage. (**a**) Evolution of *CV* in time. (**b**) Modeling *CV* using a linear regression to compute and predict the parameter for adaptive threshold.

In image processing, *CV* can be used to evaluate the dispersion of pixels values according to the histogram and it is used to analyze the evolution of biological experiments. In the confluence and apoptosis stages, the *CV* has the minimum value. Figure 6a shows the evolution in time of Dataset_1-CPT described by the *CV* parameter.

In Figure 6b, the data set $\left\{x_{i},\;y_{i}\right\}$ with i=1, …, n where n is the length of Dataset is presented. These points represent the position in a 2D space where $x_{i}$ is the number of frame and $y_{i}$ is its *CV* value. To model this coefficient, a linear regression [41] is used which is based on Equation (4):

(4) Y=mX+b

where Y is the set of predicted values, *X* is the set of real values, m is the slope and b is the intercept. Both are the adjustable parameters for regression.

#### 3.5.3. Adaptive Method and Morphological Operations

In the adaptive method, the threshold value for binarization is calculated for smaller regions. Different regions will have different threshold values. GEMA uses an adaptive method based on Gaussian and binary functions with their respective threshold values [42].

The threshold value for binary function is computed according to the evolution of the biological experiments. This parameter will take integer values in the $interval=\left[5,\;\;11\right]$ depending on the value of *CV*. Finally, the closing and dilate morphological operations are used to enlarge the boundaries of foreground regions and shrink background color holes in an image using a structuring element [37].

## 4. Results

In manual segmentation, the total segmented area (in pixels) is computed using the formula of Gaussian area [43]. The mathematical algorithm is very useful to find the area of any irregular polygon with whatever number of sides, both in the case of concave and convex polygons as shown in Equation (5):

(5) $$A=\frac{1}{2}\;\;\;\;\overset{n-1}{\underset{i=1}{\sum }}x_{i}y_{i+1}+x_{n}y_{1}\;-\overset{n-1}{\underset{i=1}{\sum }}x_{i+1}y_{i}+x_{1}y_{n}$$

where A is the area, n is the number of sides and $\left(x_{i},\;y_{i}\right)$ with i=1, …, n are the sorted vertices of the polygon.

Each image has a specific area which depends on its height and width. This area is considered the percentage of 100%. Every manually or automatically segmented image allows for computing the area of MCF7 cells detected in the image in terms of percentage as shown in Equation (6).

(6) $$P_{cells}=\frac{A_{cells}}{A_{img}}\times 100$$

where $A_{cells}$ is the area in pixels of the cell regions detected, $A_{img}$ is the total area in pixels of image and $P_{cells}$ represents the percentage of cells to describe the evolution of experiment. 

**Figure 7:**
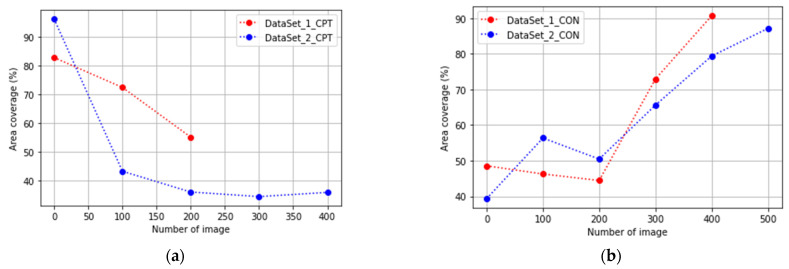
Gold Standard values: percentages of cells obtained with manual segmentation. (**a**) Evolution of cells to the confluency stage; (**b**) cells injected with CPT to generate the apoptosis stage.

In Figure 7, the evolution of the biological experiments is presented through the percentage of area occupied by cells in each image in two Datasets used to test the algorithms.

The gradient approach uses a threshold parameter with the size of 25 to binarization according to the values of the gradient image. In GEMA, images in the confluency stage can be binarized successfully with a threshold value equal to or close to 5 and images in apoptosis stages with a threshold value equal to or close to 11. Therefore, the linear regression allows for updating this value automatically when the *CV* is greater than 5%.

**Figure 8:**
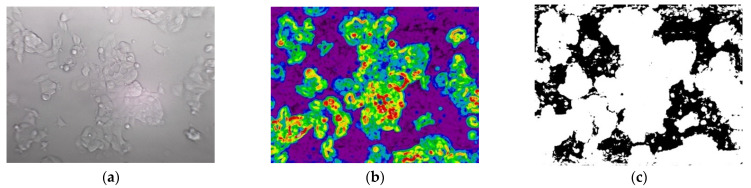
The segmentation of cells using gradient approach. (**a**) Grayscale image from Dataset_2_CONFL in apoptosis stage; (**b**) image applied morphological gradient; (**c**) binarization using a threshold value equal to 25.

**Figure 9:**
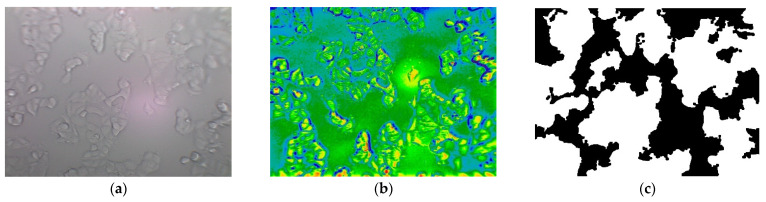
The segmentation of cells using the GEMA approach. (**a**) Grayscale image from Dataset_1_CONFL in apoptosis stage; (**b**) image applied the Gabor filter set; (**c**) binarization using a closing and dilate morphological operations.

As GEMA is an algorithm to be used in real-time experiments, the linear regression will be fitting with an error value in $range=\left[5\%,\;7\%\right]$ which allows to compute different slope and intercept values according to the evolution of biological experiment. This process is presented in Figure 6b. An example of image segmentation for both methods is presented in Figure 8 and Figure 9, respectively.

**Figure 10:**
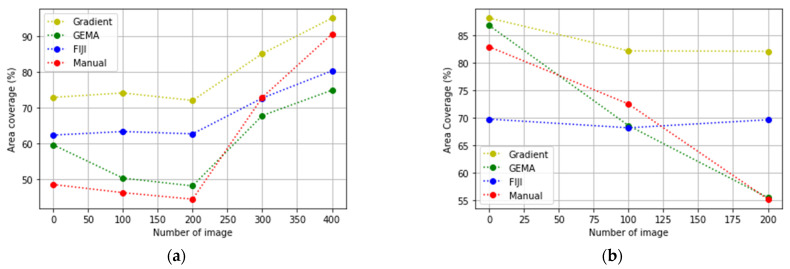
Results from Dataset_1 (**a**) The evolution to confluency stage; (**b**) the evolution to apoptosis stage.

**Figure 11:**
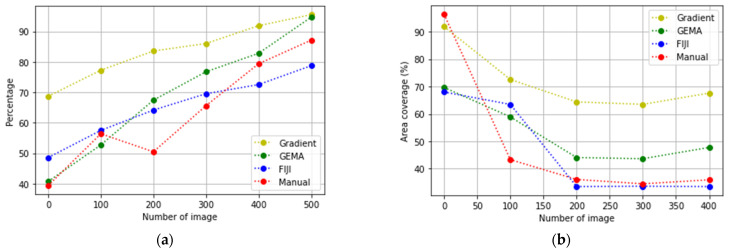
Results from Dataset_2 (**a**) The evolution to confluency stage; (**b**) the evolution to apoptosis stage.

In Figure 10 and Figure 11, the results obtained by Gradient and GEMA approaches are presented with a comparison of Manual and FIJI methods during the evaluation of two datasets. A concentration curve was performed to determine the optimal concentration and exposure time for the induction of apoptosis in the culture. Both viability and the percentage of cell death were measured using the previously described TRYPAN-BLUE methodology. Regarding the action of CTP, both in conventional plates and in microdevices, the apoptotic effect began to be seen after 2 h of exposure to the compound and its maximum effect at 18 h.

In Figure 10b, the segmentation obtained for FIJI is better than other methods. This occurs because FIJI is a semi-automatic method that includes manually setting different values to threshold parameters during the analysis of each image of the dataset according to the contrast and pixel distribution in each image. However, the accuracy evaluated in Table 1 shows that the difference between GEMA and FIJI is very low. Furthermore, in the global analysis of the results, GEMA proves to be the best algorithm for image segmentation.

According to the results, the performance of GEMA is the highest compared with other approaches. In order to evaluate GEMA, in front of gold standard values, gradient, and FIJI, two metrics are used Accuracy [44] and Dice score (also known as F1-score) [45].

Accuracy and Dice score are performance metrics for image segmentation problems. The main difference might be the fact that accuracy takes into account true negatives while the Dice score only handles true negatives as uninteresting defaults. In Equations (7) and (8), mathematical expressions for computing accuracy and Dice score are presented:

(7) $$\mathrm{Accuracy}=\frac{\mathrm{TP}+\mathrm{TN}}{n}$$

(8) $$\mathrm{Dice}\;\mathrm{score}=\frac{2\times \mathrm{TP}}{2\times \mathrm{TP}+\mathrm{FP}+\;\mathrm{FN}}$$

where TP is the among of True Positives, TN is the among of True Negatives, FP is the among of False Positives, FN is the among of False Negatives and n is the total of analyzed values.

In Table 1 and Table 2, the accuracy and Dice score values are presented which are computed by each method in front of gold standard images. These metrics have been evaluated for every 100 images in each dataset and experiment. Therefore, a total of 19 binary images is used to compute the final results by each metric. Finally, the mean of accuracy and dice score are computed to compare the performance of GEMA with FIJI and Gradient methods.

According to the results, the mean accuracy has been computed through an average of individual accuracy of the total set of segmented images by each method in front of gold standard images. Moreover, Table 1 shows that general performance of algorithms in terms of accuracy in all experiments; GEMA is about 0.86, Gradient is about 0.76 and FIJI is about 0.79.

Instead, Table 2 shows that general performance of algorithms in terms of dice score in all experiments; GEMA is about 0.86, Gradient is about 0.81 and FIJI is about 0.79.

### GEMA in Real-Time Applications

In real-time applications, a critical parameter is the time of processing. This parameter has been measured in the segmentation process by each image acquired during the biological experiments.

**Figure 12:**
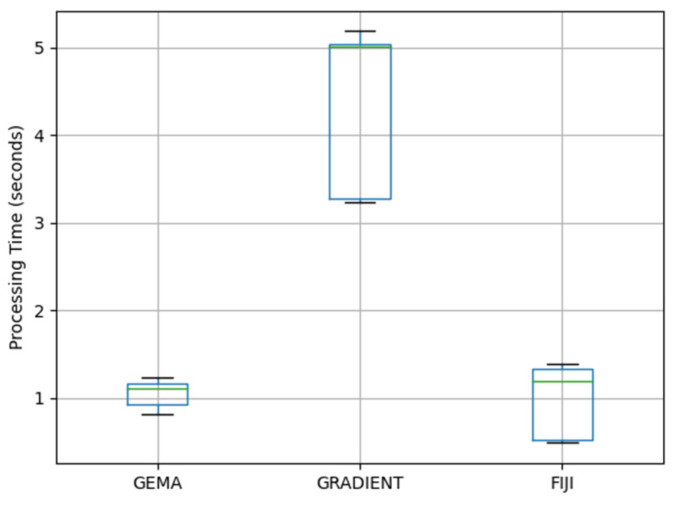
The box plots of the processing time used by each method during the segmentation process in two datasets.

In Figure 12, the box plots show the distribution of the processing time results which are used to compare and evaluate the performance of GEMA in front of the Gradient and FIJI methods.

According to these results, GEMA shows the best performance related to the Processing Time parameter with a mean value close to 1 s per image. Moreover, GEMA has the less variability, which makes it more stable in real-time applications. In Table 3, the summary of statistical parameters is presented.

To evaluate GEMA in real experiments, a GUI interface called ANSIS Real-time control interface version 0.1.2 was developed using Python version 3.8. This software was programmed by Ramiro Isa-Jara (Ecuador) and it has been shared on the GitHub platform [46]. In Table 4, the general description about inputs/outputs of ANSIS is presented.

**Figure 13:**
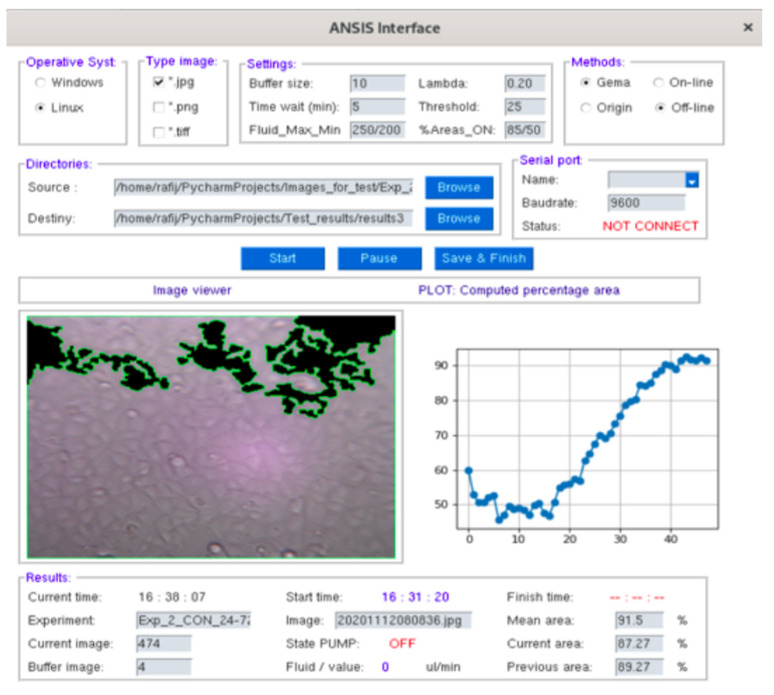
The ANSIS interface to control and analyze the evolution of biological experiment in real-time using GEMA.

In Figure 13, the ANSIS GUI is presented, which allows us to use the GEMA algorithm to detect the percentage of area occupied by MCF7 cell in real-time. Moreover, a syringe pump (Figure 1) can be controlled to inject different fluid values into a microfluid device according to increment or decrement in area of cells during the biological experiments.

## 5. Discussion and Conclusions

In this paper, a novel algorithm called GEMA for image segmentation is presented. This proposed algorithm has three main components as Gabor filter bank, the Coefficient of variation (CV), and a linear regression method.

The Gabor filter was used due to its capability in being applied in texture analysis. In biological images, it could be necessary for a frequency analysis because they could have high homogeneity in the pixel values. GEMA uses the Gabor filter bank with four directions which allow the identification of image regions with the high-frequency response and avoids redundancy in the output image. The redundancy is a relevant parameter especially in real-time algorithms.

The CV is a useful statistical measure when the dispersion should be analyzed. In this case, CV is a robust parameter because it is independent of the scale of the pixel values in each image which allows describing of the biological experiment. In the confluency and apoptosis stage, the CV has a minimum value (Figure 6a). Therefore, as the experiment progresses, the algorithm analyzes the decrease in CV to obtain the parameters of the regression model.

The last component, the linear regression method, allows for modeling of the experiment (Figure 6b.). This method is used because it is computationally efficient due to two parameters that must be computed only as the slope and intercept. It reduces the processing time focused on real-time applications. Moreover, the segmentation results in terms of biological interpretations were correctly validated by experts. Therefore, GEMA has been applied successfully in biological images to describe an experiment at a specific time. The description is performed through the segmentation of cell regions in a sequence of images taken in real-time.

According to the cell growth and development of the breast cancer line, MCF7 cells have the good performance on the plate as well as in conventional culture controls. The manufactured devices were able to maintain the cell cultures for a long period of time. The system demonstrated high performance for remote and real-time culture control, and in this context, the applications, and versatility of the system make it applicable to multiple branches of cell biology, such as development and cell differentiation, drug testing, etc.

According to the accuracy and dice mean presented in Table 1 and Table 2, *GEMA* shows the best performance compared with *FIJI* and *Gradient* methods. The average of accuracy in all the experiments is: GEMA=0.86, FIJI=0.79 and Gradient=0.76. The average of dice score in all the experiments is: GEMA=0.86, FIJI=0.79  and Gradient=0.81. These results show that the proposed method outperforms FIJI by 8% and Gradient by above 10%.

Therefore, the process described by GEMA is successfully adapted to analysis in real time of biological images. Furthermore, GEMA has the advantage of segmenting cells of the images in real-time which involves “understanding” the evolution of the biological experiment. The Coefficient of Variation allowed us to describe this evolution in time, i.e., the increase or decrease among cells in images. This parameter can be modeled using linear regression.

GEMA performs several regressions during the experiment to adjust the prediction with a maximum error of 5%. In comparison with the morphological gradient approach, GEMA does not require external parameters since it is capable to fix its behavior in order to obtain the best possible results. Finally, the computing time required by the proposed method is on average 1.05 s per image. According to Table 3, GEMA shows a stable statistical distribution. In reference to standard deviation, GEMA has 84% and 66% values better than Gradient and FIJI methods, respectively. These are main characteristics in order to apply it in real-time applications.

Future work will focus on processing different sequences of images using another drug, cell lines, or different environments to study the performance of different drug treatments in real time. Another alternative is to use another machine learning technique, such as deep learning, to improve these results.

## Figures and Tables

**Table 1:** Accuracy computed through the confusion matrix by Gradient, FIJI and GEMA approaches relative to Gold-standard images.

Dataset and Approach		Accuracy						Accuracy Mean
		0	100	200	300	400	500	
1-CPT	FIJI	0.652	0.749	0.785	-	-	-	0.73
	Gradient	0.884	0.853	0.699	-	-	-	0.81
	GEMA	0.864	0.898	0.979	-	-	-	0.91
2-CPT	FIJI	0.889	0.842	0.787	0.781	0.794	-	0.82
	Gradient	0.959	0.617	0.624	0.594	0.557	-	0.67
	GEMA	0.736	0.793	0.832	0.841	0.814	-	0.80
1-CONF	FIJI	0.796	0.816	0.806	0.732	0.616	-	0.75
	Gradient	0.733	0.711	0.704	0.841	0.918	-	0.78
	GEMA	0.816	0.871	0.898	0.901	0.821	-	0.86
2-CONF	FIJI	0.866	0.858	0.819	0.844	0.850	0.865	0.85
	Gradient	0.680	0.757	0.653	0.774	0.860	0.906	0.77
	GEMA	0.871	0.881	0.798	0.830	0.910	0.918	0.87

**Table 2:** Dice score computed through the confusion matrix by Gradient, FIJI and GEMA approaches relative to Gold-standard images.

Dataset and Approach		Dice Score						Dice Score Mean
		0	100	200	300	400	500	
1-CPT	FIJI	0.745	0.805	0.810	-	-	-	0.79
	Gradient	0.933	0.906	0.785	-	-	-	0.87
	GEMA	0.920	0.915	0.859	-	-	-	0.90
2-CPT	FIJI	0.940	0.830	0.742	0.725	0.747	-	0.80
	Gradient	0.979	0.694	0.659	0.631	0.620	-	0.72
	GEMA	0.842	0.797	0.802	0.803	0.781	-	0.80
1-CONF	FIJI	0.790	0.813	0.800	0.789	0.737	-	0.79
	Gradient	0.784	0.763	0.751	0.900	0.956	-	0.83
	GEMA	0.833	0.846	0.825	0.858	0.863	-	0.85
2-CONF	FIJI	0.844	0.872	0.838	0.882	0.902	0.918	0.88
	Gradient	0.712	0.822	0.745	0.852	0.919	0.948	0.83
	GEMA	0.842	0.893	0.829	0.882	0.945	0.955	0.89

**Table 3:** A summary of statistical measures by each method according to the processing time.

Method	Mean	Median	Standard Deviation
GEMA	1.05	1.11	0.14
Gradient	4.31	5.01	0.88
FIJI	0.95	1.20	0.41

**Table 4:** A general description of the ANSIS Real-time control interface.

Inputs/Outputs	Description	Type/Value
Operative system	To run the program	Windows, Linux (64 bits)
Image type	Read and process images	JPG, PNG, TIFF
Settings	Max fluid value to pump	250–300 μL/min
	Min fluid value to pump	200–250 μL/min
	Time to process a new image	0–5 min
Methods	Algorithms for segmentation	GEMA, Gradient
	Type of analysis	Online (real-time), Offline
Directories	Read images and save results	Source, Destiny
Serial port	Connect to syringe pump	Serial COM
	Baud rate value	9600 bpm
Graphics	Visualization	Image segmentation, Real-time of cell growth
Results	Percentage of cell area	Mean, Current, Previous
	Name of images	Image
	State of pump	ON/OFF

## Data Availability

The data described in this article are openly available in the https://www.kaggle.com/datasets/ramiroisajara/dataset-gema/ (accessed on 22 June 2022).
